# C‐type natriuretic peptide in combination with sildenafil attenuates proliferation of rhabdomyosarcoma cells

**DOI:** 10.1002/cam4.642

**Published:** 2016-01-26

**Authors:** Masahiro Zenitani, Takashi Nojiri, Shuichiro Uehara, Koichi Miura, Hiroshi Hosoda, Toru Kimura, Kengo Nakahata, Mikiya Miyazato, Hiroomi Okuyama, Kenji Kangawa

**Affiliations:** ^1^ Department of BiochemistryNational Cerebral and Cardiovascular Center Research InstituteSuita‐CityOsakaJapan; ^2^ Department of Pediatric SurgeryOsaka University Graduate School of MedicineSuita‐CityOsakaJapan; ^3^ Departments of Regenerative Medicine and Tissue EngineeringNational Cerebral and Cardiovascular Center Research InstituteSuita‐CityOsakaJapan

**Keywords:** Antiproliferation, cGMP, CNP, ERK, PDE5 inhibitor

## Abstract

Rhabdomyosarcoma (RMS) is a malignant mesenchymal tumor and the most common soft tissue sarcoma in children. Because of several complications associated with intensive multimodal therapies, including growth disturbance and secondary cancer, novel therapies with less toxicity are urgently needed. C‐type natriuretic peptide (CNP), an endogenous peptide secreted by endothelial cells, exerts antiproliferative effects in multiple types of mesenchymal cells. Therefore, we investigated whether CNP attenuates proliferation of RMS cells. We examined RMS patient samples and RMS cell lines. All RMS clinical samples expressed higher levels of guanylyl cyclase B (GC‐B), the specific receptor for CNP, than RMS cell lines. GC‐B expression in RMS cells decreased with the number of passages in vitro. Therefore, GC‐B stable expression lines were established to mimic clinical samples. CNP increased cyclic guanosine monophosphate (cGMP) levels in RMS cells in a dose‐dependent manner, demonstrating the biological activity of CNP. However, because cGMP is quickly degraded by phosphodiesterases (PDEs), the selective PDE5 inhibitor sildenafil was added to inhibit its degradation. In vitro, CNP, and sildenafil synergistically inhibited proliferation of RMS cells stably expressing GC‐B and decreased Raf‐1, Mitogen‐activated protein kinase kinase (MEK), and extracellular signal‐regulated kinase (ERK) phosphorylation. These results suggested that CNP in combination with sildenafil exerts antiproliferative effects on RMS cells by inhibiting the Raf/MEK/ERK pathway. This regimen exerted synergistic effects on tumor growth inhibition without severe adverse effects in vivo such as body weight loss. Thus, CNP in combination with sildenafil represents a promising new therapeutic approach against RMS.

## Introduction

Rhabdomyosarcoma (RMS), a malignant mesenchymal tumor, is the most common soft tissue sarcoma in children. RMS is classified histopathologically and biologically into two major types: embryonal (ERMS) and alveolar (ARMS) 1. Although clinical outcomes for RMS patients have improved over the past two decades due to advances in multimodal therapy including surgery, radiotherapy, and chemotherapy, RMS cases with high‐risk features such as relapse or systemic metastatic disease have a poor prognosis and limited treatment options 2. Furthermore, late complications including growth disturbance and secondary cancer remain important problems to be solved. Consequently, novel therapies with reduced toxicity and improved tolerability relative to conventional chemotherapy are urgently required.

C‐type natriuretic peptide (CNP) is an endogenous peptide secreted by vascular endothelial cells. The natriuretic peptide family includes atrial and brain natriuretic peptides (ANP and BNP, respectively) 3. CNP inhibits the proliferation of several types of mesenchymal cells, including vascular smooth muscle cells 4, 5, kidney mesangial cells 6, 7, 8, and fibroblasts 9, 10. However, it remains unknown whether CNP can inhibit proliferation of RMS cells.

CNP binds to and activates the guanylyl cyclase B (GC‐B) receptor, resulting in production of cyclic guanosine monophosphate (cGMP) as a second messenger and activation of downstream molecules 11. Cyclic GMP is quickly degraded to GMP by phosphodiesterases (PDEs). Because PDE5 is a major negative regulator of cGMP, PDE5 inhibitors such as sildenafil, currently used for treatment of erectile dysfunction 12 and pulmonary hypertension 13, have been used in conjunction with natriuretic peptides or other cGMP inducers to prevent degradation of cGMP by PDE5.

In this study, we demonstrated that CNP attenuates proliferation of RMS cells and that sildenafil potentiates the antiproliferative effects of CNP, and examined the underlying mechanisms responsible for this effect.

## Materials and Methods

### RMS tissue from patients

Tissue from two ERMS patients and three ARMS patients was obtained from the Department of Pediatric Surgery, Osaka University Graduate School of Medicine. Written informed consent to use resected specimens for this study was obtained from all the patients/parents/guardians, and the study was approved by the Institutional Review Board (IRB) of Osaka University Hospital (IRB number: 15022). It conforms to the provisions of the Declaration of Helsinki in 1995. Patient and sample characteristics are provided in the Table 1.

**Table 1:** Patient and sample characteristics

Case	Age	Sex	Type	Primary site	Metastasis	Follow‐up	Primary or recurrent
1	2Y11M	M	Embryonal	Prostae	–	Alive	Primary
2	9Y0M	M	Alveolar	Abdominal wall	–	Alive	Primary
3	13Y3M	M	Alveolar	Anterachial region	Axillary LN	Alive	Primary
4	3Y10M	F	Embryonal	Retroperitoneum	–	Expired	Primary
5	11M	M	Alveolar	Gastrocnemius	Retro‐peritoneum (recurrence)	Alive	Recurrence

LN, lymph node.

### Cell culture and establishment of cells stably expressing GC‐B

The human RMS cell lines RD and KYM‐1, derived from ERMS, were purchased from Japanese Collection of Research Bioresources Cell Bank (Ibaraki, Japan), RMS‐YM cells derived from ERMS were purchased from Riken Cell Bank (Tsukuba, Japan), and RH30 cells derived from ARMS were purchased from American Type Culture Collection (ATCC) (Rockville, MD). All cell lines were cultured in RPMI‐1640 medium (Life Technologies, Grand Island, NY) supplemented with 10% fetal calf serum (Invitrogen GIBCO, Tokyo, Japan) in 5% CO_2_ in air at 37°C.

The plasmid used for preparing retrovirus vector expressing GC‐B‐FLAG (pCX4 puro GC‐B‐FLAG) was constructed by introducing the GC‐B‐FLAG fragment, amplified by polymerase chain reaction (PCR) from human GC‐B obtained from Promega (Madison, WI), into the *Not*I site of pCX4 puro. The vector was transfected into BOSC cells with pE‐eco and pGp (TAKARA BIO, Kyoto, Japan) using FuGENE6 (Promega) to produce ecotropic retrovirus. After transfection, culture supernatants were collected, and filtered. Retroviral infection of RD and RMS‐YM cells was performed using the retrovirus and Ecotropic Receptor Booster (TAKARA BIO). After infection, cells were selected with puromycin. These resultant cell lines are referred to as RD‐GC‐B or RMS‐YM‐GC‐B.

### Reagents

CNP was purchased from Peptide Institute (Osaka, Japan) and sildenafil was purchased from Sigma‐Aldrich (St. Louis, MO). Antibodies were purchased as follows: antiphosphorylated extracellular‐signal‐regulated kinase (ERK)1/2 (Thr202/Tyr204), anti‐total ERK and anti‐GAPDH from Santa Cruz Biotechnology (Dallas, TX), antiphosphorylated Raf‐1 (Ser338), antiphosphorylated MEK1/2 (Ser217/221), hairy/enhancer of split (HES‐1), *β*‐catenin, GLI1, and phosphorylated Akt (Ser473) from Cell Signaling Technology (Beverly, MA), and anti‐Ki67 antibody (clone MIB‐1), as a marker for cell proliferation, from Dako Japan (Kyoto, Japan). Caspase‐3 activity was evaluated using a Nuc‐ View^™^ 488 Caspase‐3 assay kit (Biotium Inc., Cam‐ bridge, UK). The cells were incubated at room temperature for 30 min with 1 *μ*mol/L Nuc‐View 488 substrate solution.

### Gene expression analysis

Total RNA was isolated from cell lines and patient tissue samples homogenates using guanidinium‐phenol‐chloroform extraction and the RNeasy mini kit (Qiagen, Hilden, Germany). The obtained RNA was reverse‐transcribed into complementary DNA using a QuantiTect Reverse Transcription Kit (Qiagen). Quantitative PCR assays were performed using the SYBR Premix Ex Taq kit (TAKARA BIO) on a Light Cycler 480 System II (Roche Applied Science, Indianapolis, IN). The following primers were used: GC‐B, sense 5′‐TAAGAATGAGCATTACCGTACC‐3′ and antisense 5′‐GCGAGCATCCAGATACAG‐3′; 36B4, sense 5′‐TCATTGTGGGAGCAGACAATGTGGG‐3′ and antisense 5′‐AGGTCCTCCTTGGTGAACACAAAGC‐3′. Quantitation of gene expression was normalized to the housekeeping gene 36B4.

### Measurement of intracellular cyclic GMP

Rhabdomyosarcoma cells were seeded onto 24‐well plastic plates and incubated for 24 h. After the cells were confluent as a monolayer, they were washed twice with 500 *μ*L of serum‐free RPMI‐1640 and incubated in serum‐free medium for 24 h. CNP was dissolved in 5 mmol/L isobutylmethylxanthine (IBMX) at final concentrations of 1 nmol/L–10 *μ*mol/L. Various concentrations of CNP were added to the medium, and the monolayer was incubated at 37°C for an additional 15 min. The medium was rapidly removed by aspiration, and 400 *μ*L of 70% ethanol containing 100 *μ*mol/L hydrochloric acid (HCl) was added to lyse the cells. The cGMP content of the dried extract was quantitated using a radioimmunoassay (RIA) kit (Yamasa Shoyu, Tokyo, Japan).

### Cell proliferation assay

A total of 2000 cells per well were seeded into 96‐well plates in 100 *μ*L of culture medium. After 24 h, the wells were washed twice with 1% serum‐free RPMI‐1640 medium and subsequently incubated in this medium. Twenty‐four hours after serum deprivation, CNP (1 *μ*mol/L) and/or sildenafil (100 *μ*mol/L) were added, and the cells were incubated for 0–4 days. Cell proliferation was determined in sextuplicate using the Cell Counting Kit‐8 (Nacalai Tesque, Kyoto, Japan).

### Treatment of cells with CNP and sildenafil, preparation of cell lysate, and western blot analysis

A total of 1 × 10^5^ cells per well were plated in 12‐well plastic plates in complete media. Cells were serum‐starved with RPMI‐1640 medium containing 1% serum for 24 h, and then treated with CNP (1 *μ*mol/L) and/or sildenafil (100 *μ*mol/L) for indicated times. Cells were lysed with NP‐40 buffer (0.5% NP‐40, 10 mmol/L Tris [pH 7.6], 0.15 mol/L NaCl, 1.5 mmol/L MgC92) containing phosphatase inhibitor cocktail (Nacalai Tesque). Samples were electrophoresed on 4–15% gradient gels (Bio‐Rad, Hercules, CA, USA) and transferred onto polyvinylidene difluoride transfer membranes (Millipore Bedford, MA). Antibody binding was detected with an enhanced chemiluminescence detection system (Amersham Pharmacia Biotech, Piscataway, NJ).

### Xenograft efficacy studies

All animal experiments were performed according to a protocol approved by the Animal Care Ethics Committee of the National Cerebral and Cardiovascular Center Research Institute. Five‐week‐old male BALB/c nu/nu mice and 5‐week‐old male nonobese diabetic/severe combined immunodeficiency (NOD)⁄severe combined immunodeficiency (SCID) mice were purchased from Japan SLC (Shizuoka, Japan) and Oriental Yeast (Tokyo, Japan), respectively.

RD‐GC‐B cells (1–3 × 10^7^ cells) were subcutaneously implanted into the chest and abdominal area of NOD/SCID mice. When the subcutaneous tumors reached a diameter of 10–12 mm, they were resected, divided into 5‐mm pieces, and reimplanted into the chest area of BALB/c nu/nu mice. Ten days after tumor implantation, administration of CNP and/or sildenafil was initiated. CNP was continuously infused using an osmotic mini‐pump (Alzet Model 1003D; Duret Corporation, Cupertino, CA), as previously reported 14. CNP was subcutaneously administered at a rate of 2.5 *μ*g/kg/min, and treatment continued for 4 weeks. Sildenafil was intraperitoneally administered at a dose of 20 mg/kg every other day for 4 weeks. Tumor volume was calculated by the formula: V (mm^3^) = 0.5 × *a* × *b* ^2^, where *a* is the longest tumor axis and *b* is the shortest tumor axis. Upon termination of the experiment, after blood was withdrawn from the inferior vena cava, tumors were excised and weighed, and tumor samples were processed for immunohistochemical and Western blot analysis.

### Immunohistochemistry

Immunohitochemical staining was performed by the avidin‐biotin‐peroxidase complex (ABC) method using the Vectastain Elite ABC kit (Vector, Burlingame, CA) as described previously 14. Because the anti‐Ki67 antibody was mouse monoclonal antibody, a mouse on mouse immunodetection kit (Vector) was used. Images of immunohistochemical staining were captured using a FSX100 Bio Imaging Navigator microscope (Olympus, Tokyo, Japan). Ki67‐positive and ‐negative cells were counted using the CellSens Dimension software (Olympus). Ten randomly selected fields per section and 7–8 sections per animal were analyzed at 20 × magnification.

### Plasma CNP measurements

Osmotic mini‐pumps containing CNP were implanted subcutaneously in 6‐week‐old male C57BL/6 mice purchased from Japan SLC. CNP was administered at a rate of 2.5 *μ*g/kg/min, and blood samples from these treated mice and untreated mice were withdrawn 3 days after the implantation. CNP content was determined using a CNP RIA kit (Phoenix Pharmaceuticals, Belmont, CA).

### Statistical analysis

Data were analyzed using GraphPad Prism version 6.0 (GraphPad Software, San Diego, CA), and expressed as means ± standard error. Between‐group comparisons were performed using the Welch's *t* test or Mann–Whitney *U* test. Multiple‐group comparisons were performed using one‐way or two‐way ANOVA, followed by the post hoc Tukey–Kramer (pairwise comparisons) or Dunnett test (comparisons with controls). *P *<* *0.05 was considered significant.

## Results

### GC‐B expression levels in RMS cell lines are lower than those in RMS tumor samples

**Figure 1:**
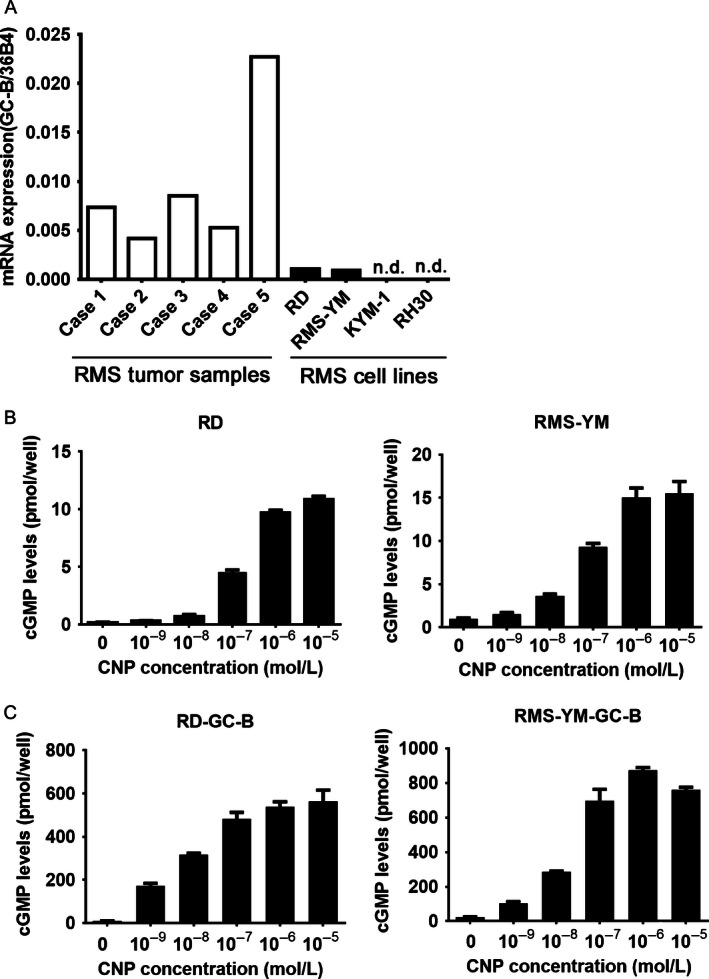
GC‐B is upregulated in rhabdomyosarcoma (RMS) tumor samples, but is expressed at significantly lower levels in RMS cell lines. (A). GC‐B mRNA levels (normalized against 36B4, used as an internal control), as determined as analyzed by quantitative PCR, in five RMS patient samples (white) (case 1 and 4: embryonal type; case 2, 3 and 5: alveolar type, case 1–4: primary tumor; case 5: recurrent tumor) and four RMS cell lines (black). (B). CNP bioactivities in RD and RMS‐YM cells, determined by measurement of intracellular cyclic guanosine monophosphate (cGMP) levels. The data represent the average from four wells with the indicated standard error. (C). CNP bioactivities in RD‐GC‐B and RMS‐YM‐GC‐B cells determined by measurement of intracellular cGMP levels.

GC‐B expressions in tumor samples of RMS patients and RMS cell lines were initially evaluated. All samples from RMS patients and two RMS cell lines, RD and RMS‐YM cells, expressed GC‐B. In contrast, KYM‐1 and RH‐30 cells had no detectable GC‐B mRNA expression (Fig. 1A). To determine whether the GC‐B receptors in RD and RMS‐YM cells were functional, we measured cGMP production in response to CNP treatment. CNP increased the intracellular cGMP levels in a dose‐dependent manner (Fig. 1B). In these cells, cGMP levels almost reached a plateau at CNP concentration of 1 *μ*mol/L (Fig. 1B). Therefore, in the following in vitro experiments, we used 1 *μ*mol/L CNP.

Next, we compared GC‐B expression levels between tumor samples from RMS patients and RMS cell lines. The RMS patient samples had 5–26‐fold higher GC‐B expression than the RD or RMS‐YM cell lines. The recurrent tumor sample (Case 5) had significantly higher GC‐B expression than the samples from primary tumors (Fig. 1A). Additionally, we noticed that the amount of GC‐B expression in RD cells decreased with the number of passages (Fig. S1), as previously reported for smooth muscle and osteoblast cells 15, 16. For these reasons, we established RD and RMS‐YM cells stably expressing GC‐B to mimic the RMS patient samples (Fig. S2). Hereafter, we refer to these cell lines as RD‐GC‐B and RMS‐YM‐GC‐B, respectively. These cells exhibited robust increases in GC‐B expression and CNP‐dependent cGMP production (Fig. 1C).

### CNP/GC‐B/cGMP system attenuates RMS cell proliferation, and sildenafil potentiates the antiproliferative effect

**Figure 2:**
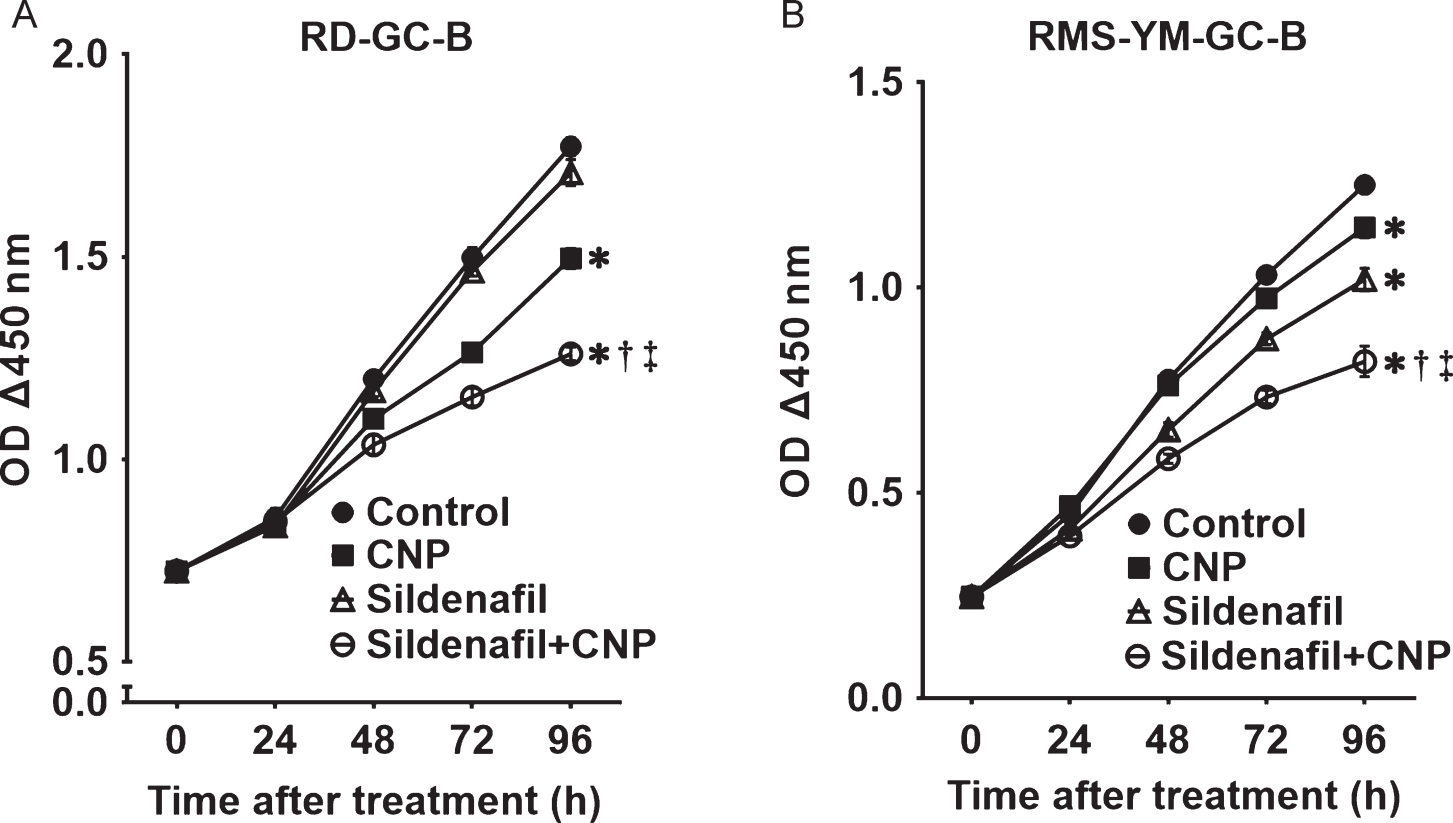
CNP inhibits rhabdomyosarcoma (RMS) cell proliferation, and sildenafil potentiates this inhibition. RD‐GC‐B (A) and RMS‐YM‐GC‐B (B) cells were incubated with solvent alone (Control), CNP (1 *μ*mol/L) alone, sildenafil (100 *μ*mol/L) alone, and the combination of sildenafil (100 *μ*mol/L) and CNP (1 *μ*mol/L). Cell proliferation was measured every 24 h using a WST‐8 assay. Data are expressed as means ± SE (*n *= 6 in each group) from three independent experiments; *P < 0.05 versus solvent alone (Control); ^†^ *P *< 0.05 versus CNP alone; ^‡^ *P *< 0.05 versus sildenafil alone.

We tested the effects of CNP/GC‐B/cGMP signaling system on proliferation of RD‐GC‐B and RMS‐YM‐GC‐B cells. As shown in Figure 2, cell proliferation was significantly attenuated in both RD‐GC‐B (Fig. 2A) and RMS‐YM‐GC‐B cells (Fig. 2B) 96 h after CNP treatment. The effects of cGMP can be enhanced and prolonged by suppressing cGMP degradation using sildenafil, a PDE5 inhibitor 25. Therefore, we examined the effects of sildenafil on cell proliferation of RMS cells by CNP. Combination treatment with sildenafil and CNP significantly decreased the proliferation of RD‐GC‐B (Fig. 2A) and RMS‐YM‐GC‐B cells (Fig. 2B) relative to treatment with CNP or sildenafil alone. Morphological appearance of cell death was not observed during 96 h in treatment with sildenafil and/or CNP. In addition, caspase‐3 activity was not significantly induced in RD‐GC‐B cells after the treatment with CNP and/or sildenafil, when we evaluated apoptosis under the same conditions of the cell proliferation assay using a Nuc‐View^™^ 488 Caspase‐3 substrate (data not shown). Together, these findings demonstrate that the CNP/GC‐B/cGMP system attenuates proliferation of GC‐B–expressing ERMS cells, and that PDE5 inhibition synergistically potentiates this antiproliferative effect.

### CNP inhibits Raf/MEK/ERK signaling, and sildenafil enhances and prolongs the inhibition

**Figure 3:**
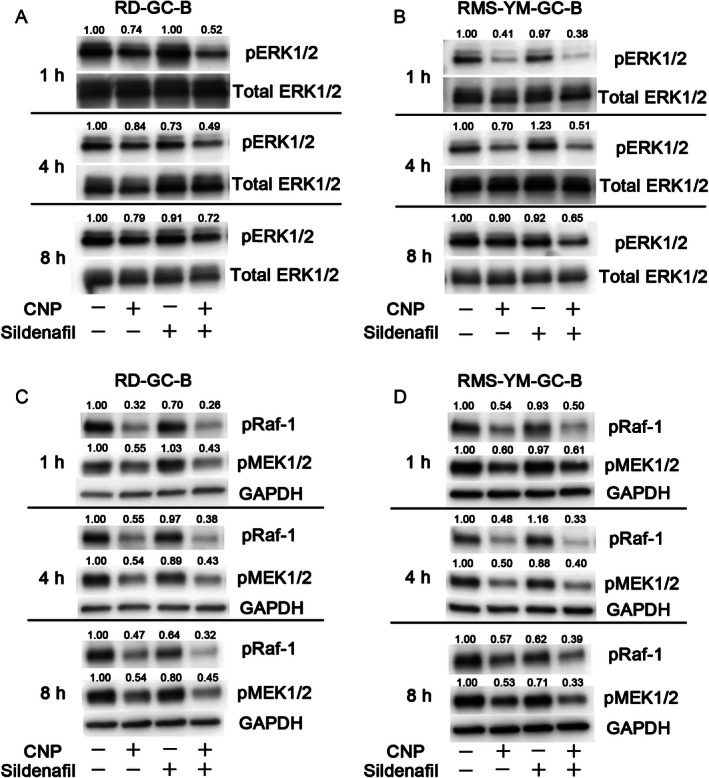
CNP inhibits Raf/MEK/ERK (extracellular signal‐regulated kinase) signaling, and sildenafil enhances and prolongs this signal inhibition. Reduced phosphorylation of Raf‐1, MEK and ERK1/2 was confirmed by western blotting under the same conditions used to observe growth inhibition. RD‐GC‐B (A) and (C) and RMS‐YM‐GC‐B (B and D) cell lysates were prepared after treatment with solvent alone (Control), CNP (1 *μ*mol/L) alone, sildenafil (100 *μ*mol/L) alone, and the combination of sildenafil (100 *μ*mol/L) and CNP (1 *μ*mol/L). Data collected after 1–8 h of treatment are presented. Data represent one of three independent experiments.

Next, we investigated the mechanism underlying inhibition of RMS cell proliferation by sildenafil and CNP. In RMS cells, several signaling pathways including Notch [17, 18], Wnt 19, Hedgehog 20, phosphoinositide 3‐kinase (PI3K)/AKT/mTOR 21, 22, and MAPK/ERK 23, 24 are involved in promoting cell proliferation. When we treated RD‐GC‐B and RMS‐YM‐GC‐B cells with CNP, we observed dephosphorylation of ERK (Figs. 3A and B, respectively). Although sildenafil alone did not cause dephosphorylate ERK during 8 h of treatment, it enhanced and prolonged the dephosphorylation of ERK by CNP (Figs. 3A and B). Dephosphorylation of Raf‐1 and MEK (the kinase upstream of ERK) by CNP was enhanced by sildenafil (Figs. 3C and D). Expression of HES‐1, *β*‐catenin, and GLI1, as well as phosphorylation of Akt, were unchanged by CNP and sildenafil treatment in RD‐GC‐B cells, suggesting that the Notch, Wnt, Hedgehog, and PI3K/AKT/mTOR signaling pathways were not regulated by CNP in RD‐GC‐B cells (Fig. S3). Additionally, CNP had no effect on proliferation or reduction in ERK phosphorylation in parental RD and RMS‐YM cells (data not shown). Overall, these data suggest that CNP/GC‐B/cGMP system attenuates the proliferation of RMS cells by inhibiting the Raf/MEK/ERK signaling pathway.

### CNP and sildenafil synergistically inhibit growth of RD‐GC‐B xenograft tumors

**Figure 4:**
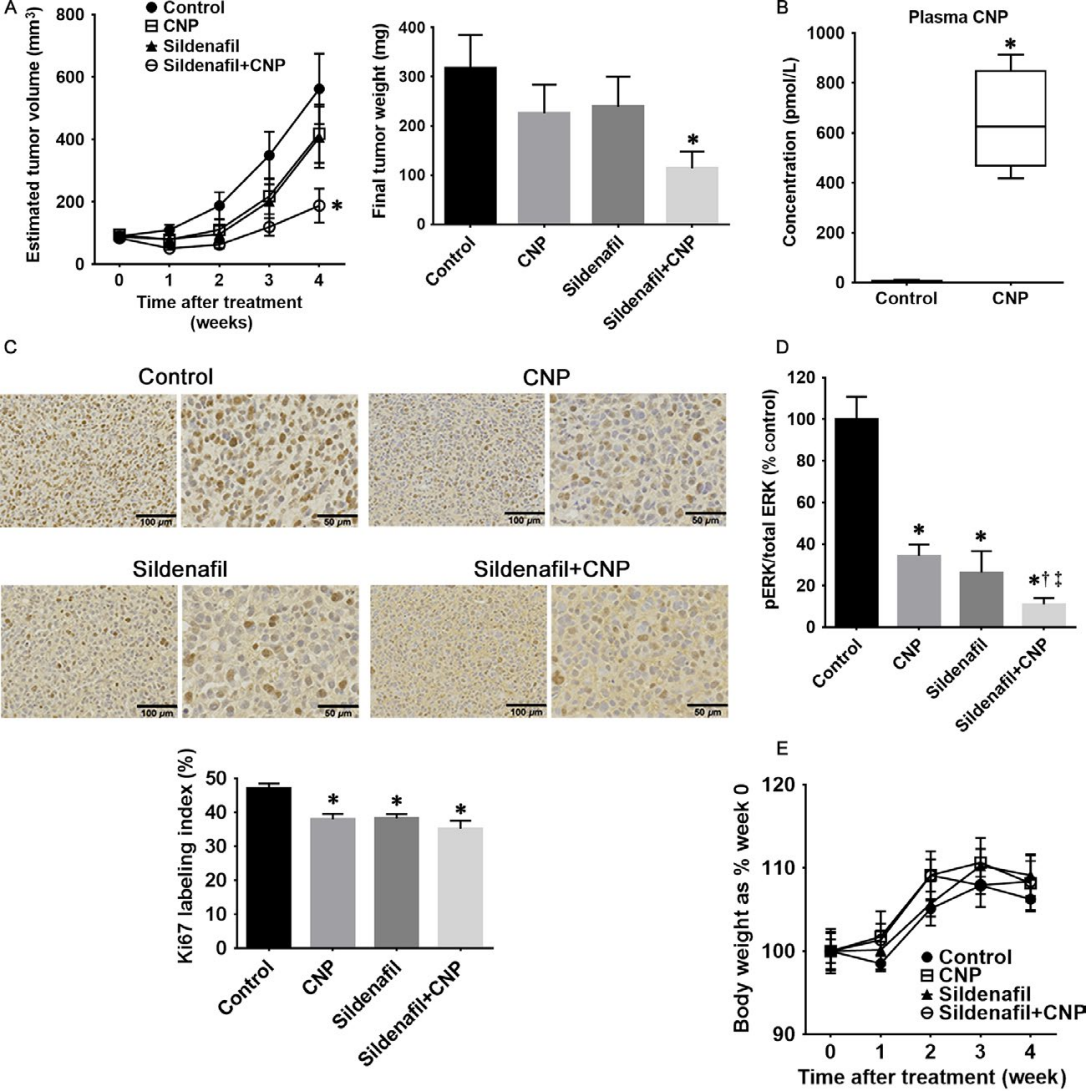
CNP and sildenafil synergistically inhibit growth of RD‐GC‐B xenograft tumors. (A). Tumor growth and harvested tumor weights of RD‐GC‐B xenografts tumors treated with vehicle control (*n* = 8), CNP (2.5 *μ*g/kg/min, continuous subcutaneous infusion, *n* = 7), sildenafil (20 mg/kg, intraperitoneal injection, every other day, *n* = 7), or a combination of sildenafil and CNP (*n* = 8). (B). Plasma CNP concentration under continuous subcutaneous CNP infusion at a rate of 2.5 *μ*g/kg/min (*n* = 5) is expressed relative to the normal control (*n* = 10). (C). Ki67 labeling index. In vivo tumor growth fraction measured by Ki67 immunohistochemical staining in RD‐GC‐B tumors in these four groups is shown as representative sections and Ki67 labeling index. (D). Phosphorylated extracellular signal‐regulated kinase (ERK) levels. Western blots of pERK1/2 versus total ERK1/2 in protein lysates from xenograft tumors in these four groups were quantitated by densitometry. Three representative blots for each group are shown in Figure S4. (E). Mice body weights in each group are expressed as % weight on week 0 during treatment. Low magnification:  × 20, scale bar 100 *μ*m (left); high magnification:  × 40; scale bar 50 *μ*m (right). Data are expressed as means ± SE (*n *=* *7–8 in each group); *P < 0.05 versus control group; ^†^ *P *< 0.05 versus CNP group; ^‡^ *P *< 0.05 versus sildenafil group.

Next, we evaluated the long‐term effects of CNP and sildenafil on tumor growth in vivo. As shown in Figure 4A, treatment with CNP or sildenafil alone did not suppress tumor growth significantly; however, the combination treatment significantly suppressed tumor growth and reduced tumor weight relative to treatment with vehicle alone (Fig. 4A). Pharmacokinetic analysis of CNP revealed that the plasma concentration of CNP was significantly increased by continuous subcutaneous infusion of CNP (Fig. 4B). Ki67 labeling index was significantly reduced in the CNP, sildenafil, and sildenafil plus CNP groups relative to the control group (Fig. 4C). Phosphorylated ERK levels were equally reduced, to almost 30% of control levels, in both the CNP and sildenafil groups, and were further reduced to almost 10% of control levels in the sildenafil plus CNP group (Fig. 4D). To evaluate tolerability, we assessed the body weights of the mice during treatment. No weight loss was observed in any of the treated groups relative to the control group (Fig. 4E).

## Discussion

Recent studies showed that several cGMP inducers regulate tumor cell proliferation. Green tea polyphenol (–)‐epigallocatechin‐3‐*O*‐gallate increases cGMP to inhibit tumor growth by activating apoptotic signaling via the Akt/eNOS/NO/cGMP/PKC*δ* pathway in multiple myeloma, gastric, pancreatic, and prostate cancers 25. The nonsteroidal anti‐inflammatory drug (NSAID) sulindac sulfide inhibits colon cancer cell growth by suppressing Wnt/*β*‐catenin signaling via the cGMP/protein kinase G (PKG) pathway 26. In this study, we examined the antiproliferative effects of CNP/GC‐B/cGMP system on RMS.

Our data demonstrate that GC‐B expression decreases with passage number in RD cells. It is the same in smooth muscle cells and osteoblasts due to oxidative stress resulting in DNA damage accumulation 16. Meanwhile, in chondrocytes, GC‐B expression increases with passage number due to the differentiation into fibroblast‐like cells 27. Therefore, they might depend on the cell types. Additionally, the differentiation may cause the higher levels of GC‐B expression in the recurrent tumor sample than in the primary tumor samples.

Phosphorylation of ERK and proliferation of two RMS cell lines were inhibited by CNP. The MAPK/ERK pathway plays a key role in promoting cell proliferation 23, 24. Activating mutations of N‐ or K‐Ras in RMS clinical samples, and in N‐Ras in the RD cell line, lead to uncontrolled cell growth 28, 29, 30. RD cells harbor the N‐Ras Q61H mutation, which constitutively activates MEK/ERK. Consistent with this, phosphorylation of ERK was detected in RD cells in the absence of stimulation (Fig. 3A). Similarly, phosphorylation of ERK was observed in unstimulated RMS‐YM cells (Fig. 3B). Although N‐Ras is active in RD cells, CNP dephosphorylated ERK, suggesting that CNP negatively regulates downstream effectors of N‐Ras such as Raf‐1 or MEK. Several studies have described the relationship between CNP and the MAPK/ERK pathway. In rat chondrosarcoma chondrocytes, fibroblast growth factor (FGF)‐2‐mediated activation of MAPK/ERK is inhibited by CNP/GC‐B/cGMP signaling at the level of Raf‐1 31. Moreover, cGMP signaling phosphorylates Raf‐1 at Ser43, resulting in the uncoupling of the Ras/Raf‐1 interaction and inactivation of the Raf/MEK/ERK pathway 32. We found that CNP attenuated phosphorylation of Raf‐1 in RMS cells (Figs. 3C and D). These findings suggest that CNP/GC‐B/cGMP signaling is a potent inhibitor of Raf/MEK/ERK pathway at the level of Raf‐1 in RMS cells.

Sildenafil synergistically potentiated the effects of CNP on cell proliferation and ERK phosphorylation in RMS, indicating that cGMP is a negative regulator of cell proliferation and ERK phosphorylation in this cancer. PDE5 is often overexpressed in various types of cancers including gastric, pancreatic, prostate, and breast cancers 25. Therefore, if both GC‐B and PDE5 are overexpressed, the combination of CNP and sildenafil may be more potent against cancer cells than normal tissue.

In tumor xenograft samples, we observed robust dephosphorylation of ERK and inhibition of tumor growth treated with sildenafil plus CNP, consistent with the results of the in vitro study. However, there is some discrepancy between in vitro and in vivo results. Although phosphorylated ERK levels were reduced by treatment of sildenafil alone in vivo, they were not reduced by sildenafil in vitro. In vivo, sildenafil was administered repeatedly; however, in vitro, RMS cells were treated with single‐dose administration. This may have caused the differences between ERK phosphorylation by sildenafil in vitro and in vivo. Additionally, there is discrepancy in the in vivo results. Although Ki‐67 labeling index and ERK‐phosphorylation were suppressed by the treatment of either CNP or sildenafil, the tumor growth was not inhibited in vivo. As shown in in vitro results, CNP significantly inhibited ERK phosphorylation in RMS cells; however, antiproliferative effects of CNP alone were relatively weak. This might be one of the causes of the discrepancy. These findings suggest that strong dephosphorylation of ERK induced by treatment with CNP and sildenafil may be required to inhibit the growth of RMS cells in vivo. Therefore, combined treatment with CNP and sildenafil represents a promising approach for therapy against RMS.

In addition to the anticancer effects of CNP and sildenafil, we evaluated the safety of the combined treatment. Side effects of intense chemotherapy, such as growth disturbance and secondary cancer, are major problems for pediatric patients with RMS; therefore, treatment regimens with a less toxic agent are desirable. CNP, an endogenous peptide, is mainly secreted by vascular endothelial cells in a wide variety of tissues, and it exerts a wide range of vasoprotective effects 14, 33, 34, 35. Moreover, we previously reported that CNP protects against cisplatin‐induced nephrotoxicity in mice via its anti‐inflammatory effects 36. Therefore, CNP is considered to be a safe alternative to other chemical compounds. Meanwhile, sildenafil is generally used to treat pulmonary arterial hypertension in children, including neonatal patients, in addition to erectile disorders in adults; in both cases, the drug lacks severe side effects 37, 38. Our results demonstrate that treatment with CNP and/or sildenafil did not induce weight loss (Fig. 4E). Therefore, the treatment with CNP and sildenafil should be well tolerated by pediatric RMS patients, and could improve their quality of life.

There are some limitations in this study. First, RMS cells stably expressing GC‐B expressed higher levels of GC‐B than clinical samples. However, given that the recurrent sample expressed a very high level of GC‐B, the findings made using RMS cells stably expressing GC‐B are still quite valuable. Although further evidence is necessary to confirm this result, it is possible that all recurrent RMS tumor cells express higher levels of GC‐B. Because there is neither a standard treatment protocol nor established evidence for therapeutic strategies against recurrent RMS, CNP/GC‐B/cGMP system represents a promising target for growth suppression of RMS cells. Second, treatment with CNP and/or sildenafil did not cause severe adverse effects such as body weight loss in vivo; however, further safety evaluation of this treatment is required. Although subcutaneous infusion of CNP at a rate of 2.5 *μ*g/kg/min does not cause hypotension 14, it should be noted that combined treatment with CNP and sildenafil may induce adverse effects, including hypotension.

In addition to the growth‐inhibitory effect of CNP, we are currently investigating whether natriuretic peptides have other anticancer effects, for example, antimetastatic effects. We recently found that ANP suppresses E‐selectin expression in vascular endothelial cells, resulting in suppression of cancer cell metastasis 39; consequently, we have initiated a multicenter randomized clinical trial to examine the use of perioperative administration of ANP to prevent cancer recurrence after lung cancer surgery. Although ANP has been well characterized as a cardiac hormone and is utilized in clinical settings, CNP has yet to be applied clinically despite abundant evidence of its role as a regulator of cardiac hypertrophy and remodeling (suggesting its use in treatment of cardiovascular disease) 40 and as a stimulator of endochondral bone growth (implying its potential value in patients with skeletal dysplasia) 41. Our findings suggest that CNP could be used clinically as an anticancer therapy.

In summary, we demonstrated that CNP in combination with sildenafil decreases ERK phosphorylation and suppresses proliferation of RMS cells. Combined treatment with CNP and sildenafil might be advantageous for RMS patients because of this regimen's lower toxicity and improved tolerability relative to common chemotherapeutic agents.

## Conflict of Interest

The authors have no conflict of interest.

## Supporting information


**Figure S1**. GC‐B mRNA expression decreases with the number of passages.

**Figure S2.** RD and RMS‐YM cells were stably expressing GC‐B.

**Figure S3.** Expression of HES‐1, *β*‐catenin, GLI1 and phosphorylation of Akt were not changed by CNP and/or sildenafil treatment in RD‐GC‐B cells.

**Figure S4.** Phosphorylated ERK levels were reduced in CNP and/or sildenafil groups, and were further reduced in the sildenafil plus CNP group than in the CNP or sildenafil groups in vivo.

## References

[1] Crist, W. M. , and L. E. Kun . 1991 Common solid tumors of childhood. N. Engl. J. Med. 324:461–471. 1988832 10.1056/NEJM199102143240706

[2] Ognjanovic, S. , A. M. Linabery , B. Charbonneau , and J. A. Ross . 2009 Trends in childhood rhabdomyosarcoma incidence and survival in the United States, 1975–2005. Cancer 115:4218–4226. 19536876 10.1002/cncr.24465 PMC2953716

[3] Sudoh, T. , N. Minamino , K. Kangawa , and H. Matsuo . 1990 C‐type natriuretic peptide (CNP): a new member of natriuretic peptide family identified in porcine brain. Biochem. Biophys. Res. Commun. 168:863–870. 2139780 10.1016/0006-291x(90)92401-k

[4] Furuya, M. , M. Yoshida , Y. Hayashi , N. Ohnuma , N. Minamino , and K. Kangawa . 1991 C‐type natriuretic peptide is a growth inhibitor of rat vascular smooth muscle cells. Biochem. Biophys. Res. Commun. 177:927–931. 1647770 10.1016/0006-291x(91)90627-j

[5] Hutchinson, H. G. , P. T. Trindade , D. B. Cunanan , C. F. Wu , and R. E. Pratt . 1997 Mechanisms of natriuretic‐peptide‐induced growth inhibition of vascular smooth muscle cells. Cardiovasc. Res. 35:158–167. 9302360 10.1016/s0008-6363(97)00086-2

[6] Canaan‐Kühl, S. , T. Ostendorf , K. Zander , K. M. Koch , and J. Floege . 1998 C‐type natriuretic peptide inhibits mesangial cell proliferation and matrix accumulation in vivo. Kidney Int. 53:1143–1151. 9573528 10.1046/j.1523-1755.1998.00895.x

[7] Osawa, H. , H. Yamabe , M. Kaizuka , N. Tamura , S. Tsunoda , and Y. Baba . 2000 C‐type natriuretic peptide inhibits proliferation and monocyte chemoattractant protein‐1 secretion in cultured human mesangial cells. Nephron 86:467–472. 11124596 10.1159/000045836

[8] Segawa, K. , K. Minami , N. Jimi , Y. Nakashima , and A. Shigematsu . 1998 C‐type natriuretic peptide inhibits rat mesangial cell proliferation by a phosphorylation‐dependent mechanism. Naunyn‐Schmiederberg's Arch. Pharmacol. 357:70–76. 10.1007/pl00005140 9459575

[9] Soeki, T. , I. Kishimoto , H. Okumura , T. Tokudome , T. Horio , K. Mori , et al. 2005 C‐Type natriuretic peptide, a novel antifibrotic and antihypertrophic agent prevents cardiac remodeling after myocardial infarction. J. Am. Coll. Cardiol. 45:608–616. 15708711 10.1016/j.jacc.2004.10.067

[10] Chrisman, T. D. , and D. L. Garbers . 1999 Reciprocal antagonism coordinates C‐type natriuretic peptide and mitogen‐signaling pathways in fibroblasts. J. Biol. Chem. 274:4293–4299. 9933630 10.1074/jbc.274.7.4293

[11] Langenickel, T. H. , J. Buttgereit , I. Pagel‐Langenickel , M. Lindner , J. Monti , K. Beuerlein , et al. 2006 Cardiac hypertrophy in transgenic rats expressing a dominant‐negative mutant of the natriuretic peptide receptor B. Proc. Natl Acad. Sci. USA 103:4735–4740. 16537417 10.1073/pnas.0510019103 PMC1450239

[12] Padma‐Nathan, H. , W. D. Steers , and P. A. Wicker . 1998 Efficacy and safety of oral sildenafil in the treatment of erectile dysfunction: a double‐blind, placebo‐controlled study of 329 patients. Sildenafil Study Group. Int. J. Clin. Pract. 52:375–379. 9894373

[13] Barst, R. J. , D. D. Ivy , G. Gaitan , A. Szatmari , A. Rudzinski , A. E. Garcia , et al. 2012 A randomized, double‐blind, placebo‐controlled, dose‐ranging study of oral sildenafil citrate in treatment‐naive children with pulmonary arterial hypertension. Circulation 125:324–334. 22128226 10.1161/CIRCULATIONAHA.110.016667

[14] Kimura, T. , T. Nojiri , H. Hosoda , S. Ishikane , Y. Shintani , M. Inoue , et al. 2015a C‐type natriuretic peptide attenuates LPS‐induced acute lung injury in mice. J. Surg. Res. 194:631–637. 25483737 10.1016/j.jss.2014.11.023

[15] Suga, S. , K. Nakao , I. Kishimoto , K. Hosoda , M. Mukoyama , H. Arai , et al. 1992 Phenotype‐related alteration in expression of natriuretic peptide receptors in aortic smooth muscle cells. Circ. Res. 71:34–39. 1318796 10.1161/01.res.71.1.34

[16] Kaneki, H. , M. Kurokawa , and H. Ide . 2008 The receptor attributable to C‐type natriuretic peptide‐induced differentiation of osteoblasts is switched from type B‐ to type C‐natriuretic peptide receptor with aging. J. Cell. Biochem. 103:753–764. 17562543 10.1002/jcb.21448

[17] Sang, L. , H. A. Coller , and J. M. Roberts . 2008 Control of the reversibility of cellular quiescence by the transcriptional repressor HES1. Science 321:1095–1100. 18719287 10.1126/science.1155998 PMC2721335

[18] Belyea, B. C. , S. Naini , R. C. Bentley , and C. M. Linardic . 2011 Inhibition of the notch‐hey1 axis blocks embryonal rhabdomyosarcoma tumorigenesis. Clin. Cancer Res. 17:7324–7336. 21948088 10.1158/1078-0432.CCR-11-1004 PMC3241994

[19] Zeng, F. Y. , H. Dong , J. Cui , L. Liu , and T. Chen . 2010 Glycogen synthase kinase 3 regulates PAX3‐FKHR‐mediated cell proliferation in human alveolar rhabdomyosarcoma cells. Biochem. Biophys. Res. Commun. 391:1049–1055. 19995556 10.1016/j.bbrc.2009.12.017 PMC2812657

[20] Tostar, U. , R. Toftgård , P. G. Zaphiropoulos , and T. Shimokawa . 2010 Reduction of human embryonal rhabdomyosarcoma tumor growth by inhibition of the hedgehog signaling pathway. Genes Cancer 1:941–951. 21779473 10.1177/1947601910385449 PMC3092259

[21] Cen, L. , F. C. Hsieh , H. J. Lin , C. S. Chen , S. J. Qualman , and J. Lin . 2007 PDK‐1/AKT pathway as a novel therapeutic target in rhabdomyosarcoma cells using OSU‐03012 compound. Br. J. Cancer 97:785–791. 17848913 10.1038/sj.bjc.6603952 PMC2360380

[22] Petricoin, E. F. , V. Espina , R. P. Araujo , B. Midura , C. Yeung , X. Wan , et al. 2007 Phosphoprotein pathway mapping: akt/mammalian target of rapamycin activation is negatively associated with childhood rhabdomyosarcoma survival. Cancer Res. 67:3431–3440. 17409454 10.1158/0008-5472.CAN-06-1344

[23] Ciccarelli, C. , F. Marampon , A. Scoglio , A. Mauro , C. Giacinti , P. De Cesaris , et al. 2005 p21WAF1 expression induced by MEK/ERK pathway activation or inhibition correlates with growth arrest, myogenic differentiation and onco‐phenotype reversal in rhabdomyosarcoma cells. Mol. Cancer. 4:41. 16351709 10.1186/1476-4598-4-41 PMC1343585

[24] Marampon, F. , G. Bossi , C. Ciccarelli , A. Di Rocco , A. Sacchi , R. G. Pestell , et al. 2009 MEK/ERK inhibitor U0126 affects in vitro and in vivo growth of embryonal rhabdomyosarcoma. Mol. Cancer Ther. 8:543–551. 19258428 10.1158/1535-7163.MCT-08-0570

[25] Kumazoe, M. , K. Sugihara , S. Tsukamoto , Y. Huang , Y. Tsurudome , T. Suzuki , et al. 2013 67‐kDa laminin receptor increases cGMP to induce cancer‐selective apoptosis. J. Clin. Invest. 123:787–799. 23348740 10.1172/JCI64768 PMC3561824

[26] Li, N. , Y. Xi , H. N. Tinsley , E. Gurpinar , B. D. Gary , B. Zhu , et al. 2013 Sulindac selectively inhibits colon tumor cell growth by activating the cgmp/pkg pathway to suppress wnt/b‐catenin signaling. Mol. Cancer Ther. 12:1848–1859. 23804703 10.1158/1535-7163.MCT-13-0048 PMC3800150

[27] Hagiwara, H. , A. Inoue , M. Furuya , S. Tanaka , and S. Hirose . 1996 Change in the expression of C‐type natriuretic peptide and its receptor, B‐type natriuretic peptide receptor, during dedifferentiation of chondrocytes into fibroblast‐like cells. J. Biochem. 119:264–267. 8882716 10.1093/oxfordjournals.jbchem.a021233

[28] Schaaf, G. , M. Hamdi , D. Zwijnenburg , A. Lakeman , D. Geerts , R. Versteeg , et al. 2010 Silencing of SPRY1 triggers complete regression of rhabdomyosarcoma tumors carrying a mutated RAS gene. Cancer Res. 70:762–771. 20068162 10.1158/0008-5472.CAN-09-2532

[29] Chardin, P. , P. Yeramian , P. Madaule , and A. Tavitian . 1985 N‐ras gene activation in the RD human rhabdomyosarcoma cell line. Int. J. Cancer 35:647–652. 3158613 10.1002/ijc.2910350513

[30] Stratton, M. R. , C. Fisher , B. A. Gusterson , and C. S. Cooper . 1989 Detection of point mutations in N‐ras and K‐ras genes of human embryonal rhabdomyosarcomas using oligonucleotide probes and the polymerase chain reaction. Cancer Res. 49:6324–6327. 2680062

[31] Krejci, P. , B. Masri , V. Fontaine , P. B. Mekikian , M. Weis , H. Prats , et al. 2005 Interaction of fibroblast growth factor and C‐natriuretic peptide signaling in regulation of chondrocyte proliferation and extracellular matrix homeostasis. J. Cell Sci. 118:5089–5100. 16234329 10.1242/jcs.02618

[32] Suhasini, M. , H. Li , S. M. Lohmann , G. R. Boss , and R. B. Pilz . 1998 Cyclic‐GMP‐dependent protein kinase inhibits the Ras/Mitogen‐activated protein kinase pathway. Mol. Cell. Biol. 18:6983–6994. 9819386 10.1128/mcb.18.12.6983 PMC109281

[33] Itoh, T. , N. Nagaya , S. Murakami , T. Fujii , T. Iwase , H. Ishibashi Ueda , et al. 2004 C‐type natriuretic peptide ameliorates monocrotaline‐induced pulmonary hypertension in rats. Am. J. Respir. Crit. Care Med. 170:1204–1211. 15333333 10.1164/rccm.200404-455OC

[34] Obata, H. , B. Yanagawa , K. Tanaka , S. Ohnishi , M. Kataoka , Y. Miyahara , et al. 2007 CNP infusion attenuates cardiac dysfunction and inflammation in myocarditis. Biochem. Biophys. Res. Commun. 356:60–66. 17336931 10.1016/j.bbrc.2007.02.085

[35] Schachner, T. , Y. Zou , A. Oberhuber , T. Mairinger , A. Tzankov , G. Laufer , et al. 2004 Perivascular application of C‐type natriuretic peptide attenuates neointimal hyperplasia in experimental vein grafts. Eur. J. Cardiothorac. Surg. 25:585–590. 15037276 10.1016/j.ejcts.2003.07.013

[36] Kimura, T. , T. Nojiri , H. Hosoda , S. Ishikane , Y. Shintani , M. Inoue , et al. 2015b Protective effects of C‐type natriuretic peptide on cisplatin‐induced nephrotoxicity in Mice. Cancer Chemother. Pharmacol. 75:1057–1063. 25814217 10.1007/s00280-015-2734-7

[37] Siehr, S. L. , E. K. McCarthy , M. T. Ogawa , and J. A. Feinstein . 2015 Reported sildenafil side effects in pediatric pulmonary hypertension patients. Front. Pediatr. 3:12. 25806361 10.3389/fped.2015.00012 PMC4353247

[38] Sabri, M. R. , and E. Beheshtian . 2014 Comparison of the therapeutic and side effects of tadalafil and sildenafil in children and adolescents with pulmonary arterial hypertension. Pediatr. Cardiol. 35:699–704. 24253611 10.1007/s00246-013-0840-z

[39] Nojiri, T. , H. Hosoda , T. Tokudome , K. Miura , S. Ishikane , K. Otani , et al. 2015 Atrial natriuretic peptide prevents cancer metastasis through vascular endothelial cells. Proc. Natl Acad. Sci. USA 112:4086–4091. 25775533 10.1073/pnas.1417273112 PMC4386325

[40] Del Ry, S. 2013 C‐type natriuretic peptide: a new cardiac mediator. Peptides 40:93. 23262354 10.1016/j.peptides.2012.12.010

[41] Yasoda, A. , and K. Nakao . 2010 Translational research of C‐type natriuretic peptide (CNP) into skeletal dysplasias. Endocr. J. 57:659–666. 20567091 10.1507/endocrj.k10e-164

