# Supplementary material for: C‐type natriuretic peptide in combination with sildenafil attenuates proliferation of rhabdomyosarcoma cells
Source: Cancer Med. 2016 Jan 26;5(5):795–805. doi: 10.1002/cam4.642 (PMC4864809; doi:10.1002/cam4.642)
Supplement: Supplementary file 1 — Figure S1. GC‐B mRNA expression decreases with the number of passages. [file CAM4-5-795-s001.docx]

**Figure S1. GC-B mRNA expression decreases with the number of passages.** Quantitative RT-PCR analysis of GC-B (normalized against 36B4) in RD cells at passage 5, 15, and 25. *, *P* < 0.05 vs. passage 25.
