# Supplementary material for: C‐type natriuretic peptide in combination with sildenafil attenuates proliferation of rhabdomyosarcoma cells
Source: Cancer Med. 2016 Jan 26;5(5):795–805. doi: 10.1002/cam4.642 (PMC4864809; doi:10.1002/cam4.642)
Supplement: Supplementary file 2 — Figure S2. RD and RMS‐YM cells were stably expressing GC‐B. [file CAM4-5-795-s002.docx]

**Figure S2. RD and RMS-YM cells were stably expressing GC-B. ~~A,~~** Quantitative RT-PCR analysis of GC-B (normalized against 36B4) shows that GC-B expression in RD-GC-B and RMS-YM-GC-B cells was increased almost 380-fold relative to RD cells (**, *P*< 0.01) and almost 150-fold relative to RMS-YM cells (*, *P*< 0.05). Significance of these differences was evaluated using Welch’s t test. **~~B,~~** ~~CNP bioactivities in RD-GC-B and RMS-YM-GC-B cells were determined by measuring intracellular cGMP accumulation.~~
