# Supplementary material for: C‐type natriuretic peptide in combination with sildenafil attenuates proliferation of rhabdomyosarcoma cells
Source: Cancer Med. 2016 Jan 26;5(5):795–805. doi: 10.1002/cam4.642 (PMC4864809; doi:10.1002/cam4.642)
Supplement: Supplementary file 3 — Figure S3. Expression of HES‐1, β‐catenin, GLI1 and phosphorylation of Akt were not changed by CNP and/or sildenafil treatment in RD‐GC‐B cells. [file CAM4-5-795-s003.docx]

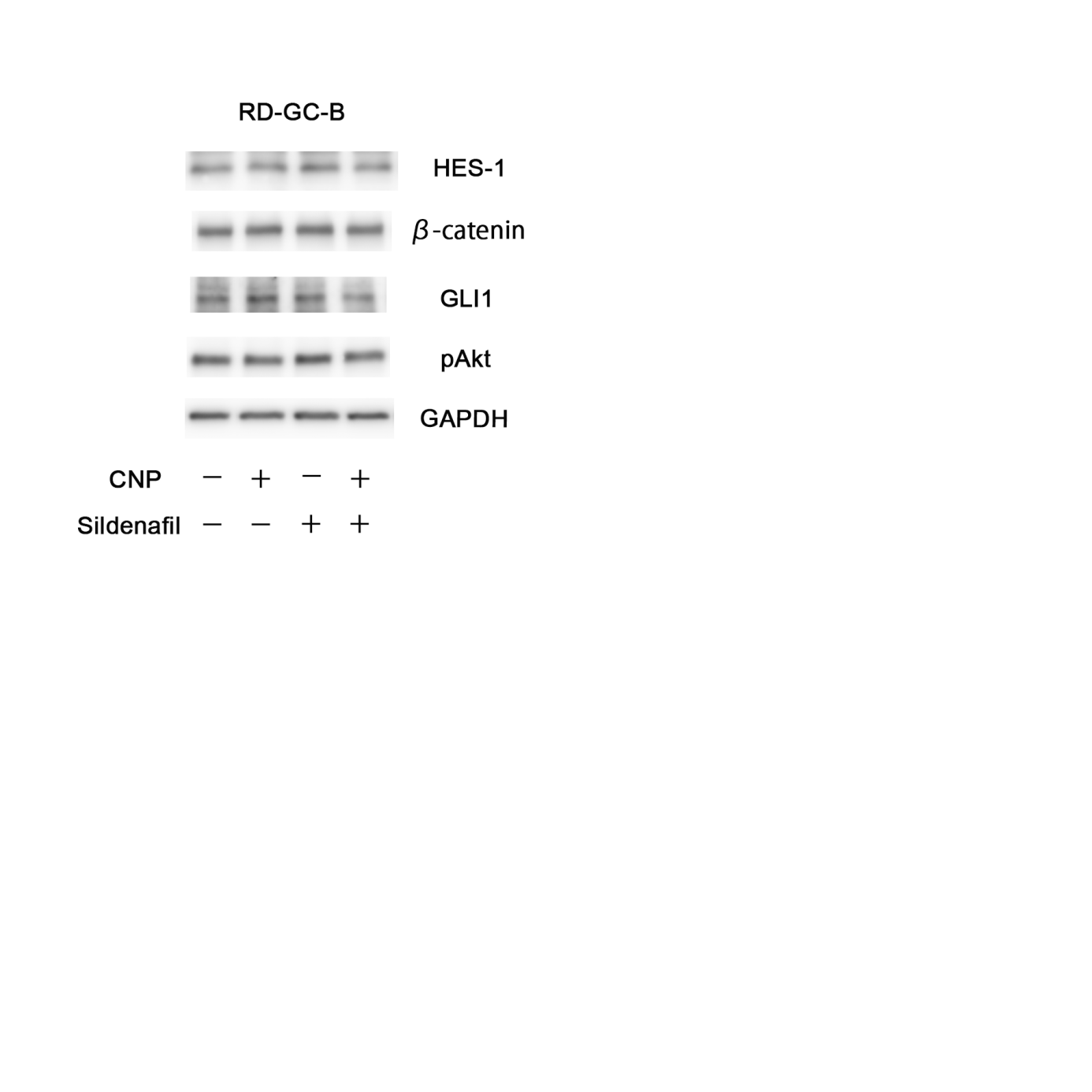


**Figure S3. Expression of HES-1, β-catenin, GLI1 and phosphorylation of Akt were unchanged by CNP and sildenafil treatment in RD-GC-B cells.** RD-GC-B cell lysates were prepared after treatment with solvent alone (Control), CNP (1 µM) alone, sildenafil (100 µM) alone, and the combination of sildenafil (100 µM) and CNP (1 µM). Data collected after 4 hours of treatment are presented.
