# Supplementary material for: C‐type natriuretic peptide in combination with sildenafil attenuates proliferation of rhabdomyosarcoma cells
Source: Cancer Med. 2016 Jan 26;5(5):795–805. doi: 10.1002/cam4.642 (PMC4864809; doi:10.1002/cam4.642)
Supplement: Supplementary file 4 — Figure S4. Phosphorylated ERK levels were reduced in CNP and/or sildenafil groups, and were further reduced in the sildenafil plus CNP group than in the CNP or sildenafil groups in vivo. [file CAM4-5-795-s004.docx]

**Figure S4. Phosphorylated ERK levels were reduced in CNP and/or sildenafil groups, and were further reduced in the sildenafil plus CNP group than in the CNP or sildenafil groups *in vivo*.** Three representative blots for each group are shown.
